# Clinical significance of persistent C4d deposition on sequential protocol biopsies in ABO-incompatible kidney transplantation

**DOI:** 10.3389/fneph.2026.1940137

**Published:** 2026-10-06

**Authors:** Yu Kijima, Hideki Ishida, Takafumi Yagisawa, Toshihito Hirai, Kohei Unagami, Tomokazu Shimizu, Toshio Takagi

**Affiliations:** 1 Department of Urology, Tokyo Women’s Medical University Hospital, Shinjuku-ku, Tokyo, Japan; 2 Department of Organ Transplant Medicine, Tokyo Women’s Medical University Hospital, Shinjuku-ku, Tokyo, Japan

**Keywords:** ABO-incompatible kidney transplantation, accommodation, C4d, microvascular inflammation, protocol biopsy

## Abstract

**Introduction:**

C4d deposition is common after ABO-incompatible (ABOi) kidney transplantation and, unlike ABO-compatible grafts, is not necessarily indicative of antibody-mediated injury.

**Methods:**

Between 2015 and 2022, 254 ABOi kidney transplantations were performed at our center, and 69 recipients with paired 3-month and 1-year protocol biopsies were classified as persistently C4d-positive (n = 54) or C4d-negative/nonpersistent (n = 15). Pre-transplant background, cellular infiltration, chronic Banff lesions according to the Banff 2019 classification, graft function, and graft survival were compared.

**Results:**

Baseline anti-A/B titers, ABO combination, donor age, and donor relationship did not differ between the groups, whereas donor sex differed significantly (P = 0.014). Microvascular inflammation, defined as glomerulitis plus peritubular capillaritis ≥2, was observed in 6/54 recipients (11.1%) versus 1/15 (6.7%), and chronic changes, defined as interstitial fibrosis score ≥1, were observed in 27/54 (50.0%) versus 6/15 (40.0%); none of these differences reached statistical significance. Graft function and survival also did not differ significantly, although all four graft losses occurred in the persistent C4d-positive group. Three of these losses were attributable to chronic active antibody-mediated rejection, diagnosed at the 1-year biopsy in one recipient and later in two recipients.

**Discussion:**

Given the selected cohort and limited statistical power, these findings represent a failure to detect an association rather than evidence of equivalence. They are consistent with accommodation but require confirmation in larger, less-selected cohorts.

## Introduction

ABO-incompatible (ABOi) kidney transplantation was introduced as a solution to the organ shortage problem in 1989 and has since been performed at our center in more than 1,000 recipients, with outcomes comparable to those of ABO-compatible kidney transplantation (1–5). However, desensitization prior to ABOi kidney transplantation has been reported to increase the risk of severe complications, particularly infectious disease (4, 5). Paired kidney exchange programs are an alternative approach, particularly in the United States. We believe that inappropriate and excessive immunosuppression is largely responsible for the increased incidence of infections in ABOi graft recipients.

A distinctive feature of ABOi kidney allografts is the frequent deposition of the complement split product C4d, a degradation fragment of C4b generated through the activation of the classical complement pathway, in the peritubular capillaries of more than half of the recipients (6, 7). In ABO-compatible kidney transplantation, peritubular capillary C4d deposition following the binding of anti-human leukocyte antigen (HLA) donor-specific antibodies (DSAs) is recognized as a histological hallmark of antibody-mediated injury and poor prognosis. In contrast, in ABOi kidney transplantation, C4d is deposited in most grafts that function well for years, a phenomenon attributed to endothelial accommodation (6, 8). Therefore, the clinical significance of C4d deposition remains unclear.

An additional clinical concern is that immediate graft loss due to hyperacute antibody-mediated rejection (ABMR) is still occasionally encountered in ABOi kidney transplantation despite adequate desensitization. In recipients at our center, assessment of the classical complement pathway using the C1q assay, together with C4d staining of graft tissue, suggests that excessive complement activation may contribute to this phenomenon (9, 10).

Most previous studies on ABOi kidney transplantation evaluated C4d at a single time point. As a single determination may not reflect a sustained process, C4d positivity was defined by its persistence across two sequential post-transplant protocol biopsies obtained at 3 months and 1 year. This study aimed to clarify whether persistent C4d deposition is associated with cellular infiltration, microvascular inflammation (MVI), the development of chronic histological lesions, graft function, and graft survival in ABOi kidney allograft recipients.

## Materials and methods

### Study design and patients

Overall, 254 ABOi kidney transplantations were performed between 2015 and 2022, followed by protocol biopsies at 3 months and 1 year postoperatively. The exclusion criteria were as follows: insufficient pathological data (n = 96; n = 91 because paired biopsies were performed outside the intended 3-month/1-year protocol windows, including scheduling delays and COVID-19-related interruptions, and n = 5 because the biopsy procedure was declined), intravenous immunoglobulin (IVIG) therapy at induction (n = 37) which may influence C4d deposition, rejection episodes requiring treatment before protocol biopsy (n = 19), thrombotic microangiopathy (TMA) (n = 12), history of transplantation (second or third transplantation, n = 11), recurrent glomerular disease including recurrent IgA severe nephropathy (n = 4) and other recurrent glomerular diseases (n = 3), postoperative severe complications like covid viral infection (n = 2), hyperacute rejection (n = 1) (**Figure 1**).

**Figure 1:**
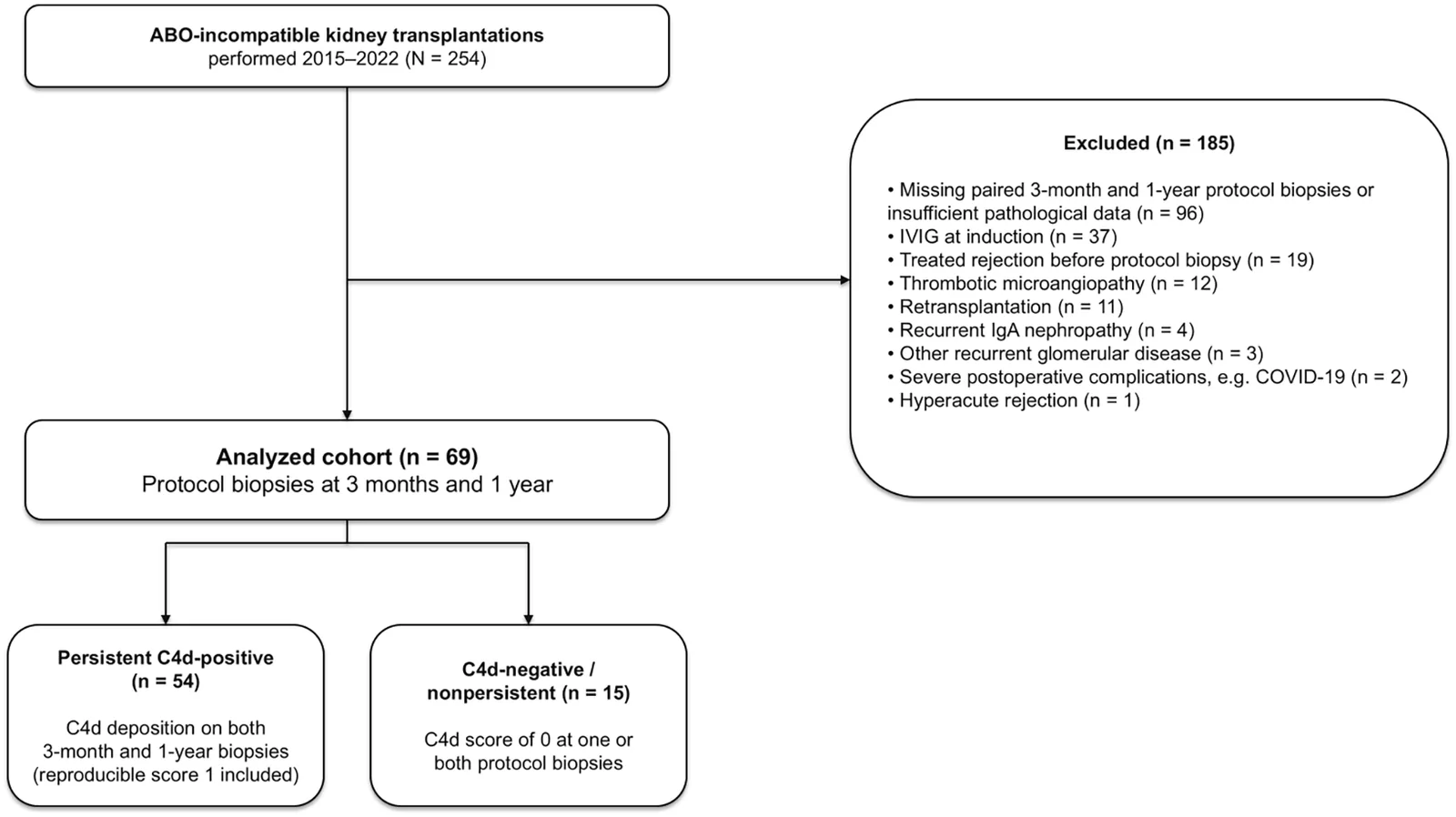
Patient flow. Among ABO-incompatible kidney transplant recipients treated between 2015 and 2022 (n = 254), recipients were classified as persistent C4d-positive (n = 54; C4d deposition present at both the 3-month and 1-year protocol biopsies, including reproducible score 1 staining at both time points) or C4d-negative/nonpersistent (n = 15; all remaining patterns, including a C4d score of 0 at either time point or positivity at only one of the two time points).

Subsequently, based on the presence or absence of persistent C4d deposition as assessed from sequential protocol biopsies performed at 3 months and 1 year post-transplantation in accordance with the protocol, the patients were divided into two groups: the persistent C4d-positive group (n = 54) and the C4d-negative/nonpersistent group (n = 15).

This study was exploratory in design, without a single pre-specified primary endpoint, and evaluated the associations between persistent C4d deposition on sequential protocol biopsies and multiple outcomes, including rejection, Banff scores, *de novo* DSAs, graft function, and graft loss. The study examined the relationship between C4d deposition and cellular infiltration scores, including interstitial inflammation (i), tubulitis (t), intimal arteritis (v), glomerulitis (g), and peritubular capillaritis (ptc), according to the Banff 2019 classification. Moreover, graft function and graft survival were analyzed in relation to these pathological changes and baseline recipient characteristics, including pre-transplant anti-A/B antibody titers.

This study was conducted in accordance with the Declaration of Helsinki, approved by the Institutional Review Board of Tokyo Women’s Medical University (Approval No. 4460-R), and granted comprehensive approval covering all patients at their first clinic visit. Written informed consent was obtained from all the participants.

### Immunosuppression protocol in ABOi kidney transplantation

The immunosuppressive regimen used in this study has been described in detail previously (2, 3). Briefly, the patients received a triple-drug immunosuppressive protocol consisting of tacrolimus, mycophenolate mofetil (MMF: at an initial dose of 1000 mg orally twice daily for the first 2 weeks, and then 750 mg twice daily), and methylprednisolone (500 mg intraoperatively, followed by tapering of the dose to 8 mg by 3 months post-transplant). The tacrolimus dose was adjusted to achieve trough levels of 8–12 ng/mL (IMX whole blood assay) during the first month after transplantation and 7–9 ng/mL over the next 2 months. Since 2001, we have been applying an immunosuppressive regimen using tacrolimus, MMF, and methylprednisolone, starting 7 days before transplantation. Induction with an anti-IL-2 receptor antibody (basiliximab, 20 mg on days 0 and 4) was performed in all ABOi recipients (**Figure 2**).

**Figure 2:**
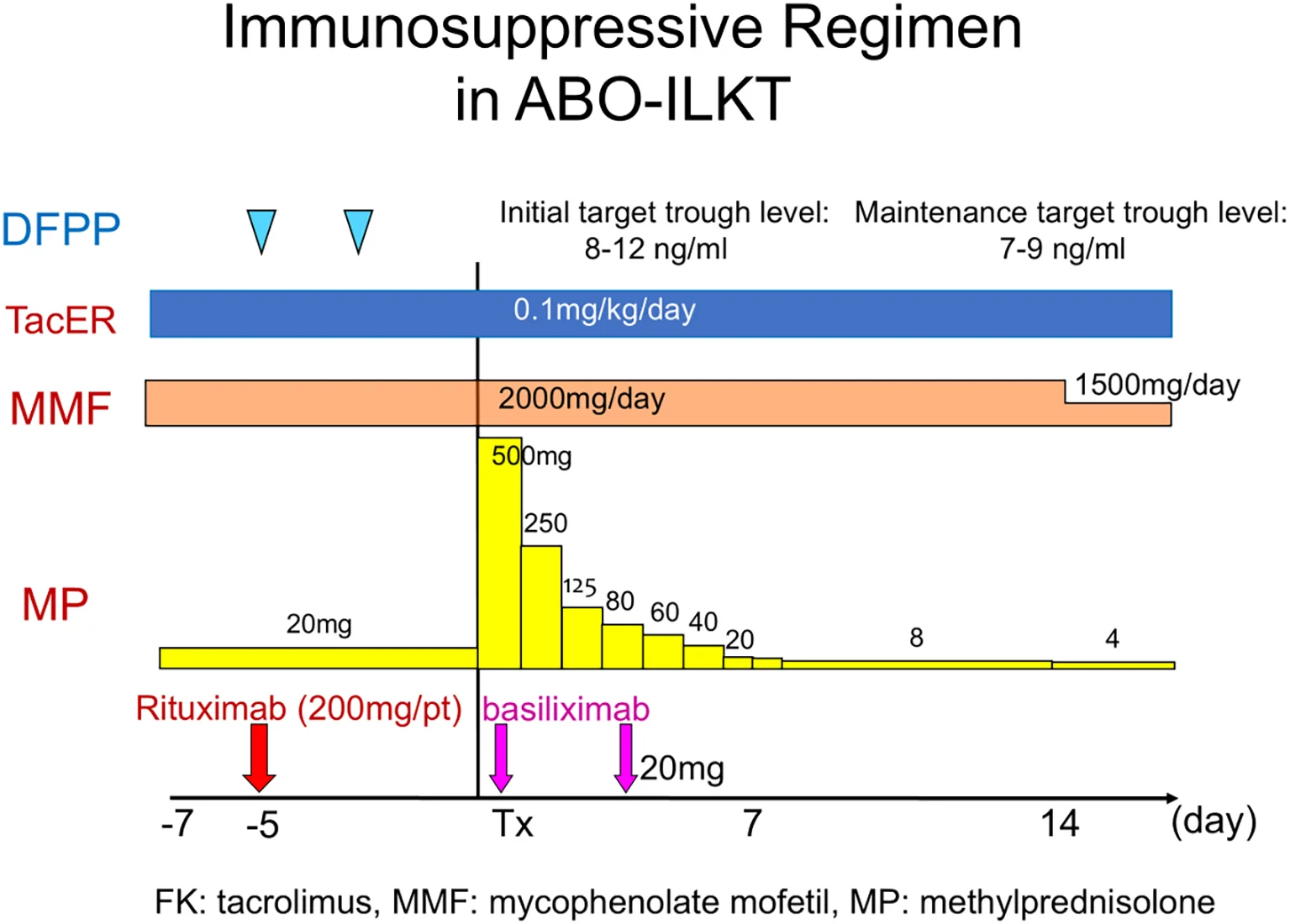
Desensitization and immunosuppression regimen used in ABO-incompatible living kidney transplantation. DFPP, double-filtration plasmapheresis; TacER, extended-release tacrolimus; MMF, mycophenolate mofetil; MP, methylprednisolone; Tx, transplantation.

Furthermore, ABOi transplant recipients underwent three or four sessions of double-filtration plasmapheresis before transplantation to remove anti-A/B antibodies until their anti-A IgG/IgM and/or anti-B IgG/IgM titers decreased to 1:32 or below. IgM anti-A and -B levels were determined using the saline agglutination technique, and the indirect Coombs test was used to measure IgG titers. Moreover, all patients received rituximab (200 mg/body) for B-cell depletion. At our center, rituximab has been used instead of splenectomy since 2004 (11). Recipients who received IVIG at induction were excluded from the present analysis because IVIG may inactivate the activated complement, thereby influencing C4d deposition (9, 10).

### Protocol biopsies

According to the protocol, renal allograft biopsies were obtained at time zero (back-table biopsy), 3 months, and 1 year after transplantation. An adequate biopsy was defined as a specimen that included seven or more glomeruli and at least one arterial cross-section. Two core biopsies were performed under ultrasound guidance using a 16-gauge biopsy gun. One core was fixed in 10% formalin and embedded in paraffin, and the other core was snap-frozen. The former was processed for routine histological analyses. Serial 3-μm thick tissue sections were stained with hematoxylin and eosin, Masson trichrome, periodic acid-Schiff, and periodic acid-methamine silver. The light microscopy findings were evaluated by a renal pathologist (YY) and scored according to the Banff classification applicable at the time of pathological assessment. Banff lesion scores were reviewed and reported to clarify the histological basis of each diagnosis.

### C4d staining in the peritubular capillaries

C4d staining was routinely performed in all renal allograft biopsies by indirect immunofluorescence on cryostat sections using a mouse monoclonal anti-human C4d antibody (Quidel, San Diego, CA, USA) at 1:40 dilution, followed by fluorescein isothiocyanate-conjugated goat anti-mouse IgG. C4d staining was scored from 0 to 3 according to the Banff criteria: C4d0, no staining (0%); C4d1, minimal staining (>0 but <10%); C4d2, focal staining (10–50%); and C4d3, diffuse staining (>50%) of the peritubular capillaries and medullary vasa recta. Pre-transplant biopsies were also examined as a 0-hour biopsy on the back table using immunofluorescence microscopy.

The persistent C4d-positive group comprised recipients with C4d deposition in both the 3-month and 1-year protocol biopsies, including recipients with a reproducible staining score of 1 (low-grade/focal) at both time points. This inclusive definition was used as the primary definition because the objective of this study was to evaluate the persistence of any detectable C4d deposition across sequential biopsies, rather than to apply Banff-defined C4d positivity as a criterion for ABMR; because the biological significance of low-grade staining may nonetheless differ from that of diffuse C4d deposition, these cases were interpreted cautiously, and a stricter, Banff-concordant definition (C4d score ≥ 2 at both time points) was evaluated separately as a sensitivity analysis. All the remaining patterns, including positivity at only one of the two time points, were classified as C4d-negative/nonpersistent. The complete transition matrix of C4d scores at 3 months and 1 year for all 69 recipients, along with the classification resulting from each definition, is provided in **Supplementary Table S1**.

### Pathological assessment

Biopsies were performed at time zero, 3 months, and 1 year in accordance with the protocol. All the biopsy specimens were evaluated according to the Banff 2019 classification criteria. The evaluated pathological findings included the classification of acute and chronic rejection reactions (T-cell-mediated rejection [TCMR], ABMR, and chronic active antibody-mediated rejection [cABMR]). C4d staining was performed on all biopsy specimens using immunofluorescence, and the results were scored according to the Banff criteria. The histological injury scores evaluated included cellular infiltration scores (i, t, g, and ptc) and chronic injury scores (interstitial fibrosis [ci], tubular atrophy [ct], vascular intimal thickening [cv], and arteriolar hyalinosis [ah]) as defined by the Banff classification. MVI was assessed as the sum of the scores for g and ptc. ABMR and cABMR were diagnosed according to Banff criteria. Anti-A/B isoagglutinins were not used to satisfy the serological criteria for ABMR, and anti-HLA DSAs were used for this purpose. ABMR diagnoses were adjudicated independently of persistent C4d group assignment, and the persistent C4d group assignment itself was not used to establish the ABMR diagnosis.

### Clinical and laboratory data

The baseline characteristics included the recipient’s age, sex, baseline ABO antibody titers (IgM and IgG), MFI values for HLA class I and class II DSAs, follow-up duration, donor age, sex, and relationship with the recipient (parent, sibling, child, spouse, or other non-blood relative). ABO antibody titers were measured prior to transplantation. DSAs were evaluated using a single-antigen bead assay (LABScreen Single Antigen, One Lambda) with a positivity threshold of MFI > 1,000 and EDTA treatment to mitigate the prozone effect. Post-transplant DSA testing was not performed at a uniform time point but at clinically indicated intervals until March 2018, when protocol-driven surveillance was progressively introduced, and available post-transplant measurements in this cohort were obtained between approximately 4 months and 3 years after transplantation. Therefore, *de novo* DSA was evaluated as detection at any point during follow-up rather than at a fixed time point. Recipients were considered evaluable if a DSA result (positive or negative) was available at any post-transplant time point. Graft function was evaluated based on serum creatinine (sCr) levels and estimated glomerular filtration rate (eGFR) at 1 year post-transplantation. eGFR was calculated using the Japanese Society of Nephrology equation. Graft loss was defined as a return to dialysis.

### Statistical analysis

Continuous variables were summarized as mean ± standard deviation or median with either interquartile range or full range, as specified for each variable in **Table 1**, depending on the distribution of the data, and compared between the C4d-positive and C4d-negative groups using the independent samples *t*-test or the Mann–Whitney *U* test, respectively. Categorical variables were summarized as numbers (%) and compared using Fisher’s exact test for 2 × 2 tables, and the distribution of ABO donor-recipient combinations (6 categories) was compared using the Fisher-Freeman-Halton exact test, as several expected cell counts were below 5, precluding a valid chi-square approximation. Graft survival was estimated using the Kaplan–Meier method and compared between groups using the log-rank test. In the primary survival analysis, time was measured from the date of transplantation. Because the persistent C4d group assignment required completion of the 1-year protocol biopsy, a landmark analysis was additionally performed with the time origin at the 1-year protocol biopsy to address the guarantee-time bias. Graft loss was defined as a return to dialysis. Death with a functioning graft was treated as a censoring event, and no such deaths occurred during the follow-up. Statistical significance was defined as a two-sided P value of < 0.05. All statistical analyses were performed using Python (version 3.12.13) with Pandas and SciPy libraries.

**Table 1:** Baseline characteristics and outcomes.

Variable	Persistent C4d-positive (n = 54)	C4d-negative/nonpersistent (n = 15)	P
Sex, male/female	37/17	11/4	1.000
Age, years (mean ± SD)	53.1 ± 11.4	51.7 ± 9.6	0.660
Donor age, years (mean ± SD)	60.3 ± 10.2	59.4 ± 8.2	0.765
Donor sex, male/female	22/32	1/14	0.014
Donor relationship, related/unrelated**	18/36	5/10	1.000
Donor eGFR, mL/min/1.73m^2^ (median [IQR])	74.2 [68.0–80.2]	71.0 [66.7–78.2]	0.383
Recipient blood type, A/B/O	13/13/28	3/4/8	—
Donor blood type, A/B/AB	23/23/8	9/4/2	—
ABO combination***	see footnote	see footnote	0.664
Follow-up, years (median [range]), from transplantation to last follow-up	4.8 [1.0–10.8]	4.1 [1.0–9.1]	0.959
Pre-transplant anti-A/B IgM titer (median [range])	16.0 [4.0–16.0]	16.0 [4.0–32.0]	0.824
Pre-transplant anti-A/B IgG titer (median [range])	8.0 [1.0–32.0]	4.0 [1.0–32.0]	0.988
Pre-existing DSA, n (%)	6 (11.1)	1 (6.7)	1.000
Serum creatinine at 1 year, mg/dL (median [IQR])	1.26 [1.02–1.56]	1.35 [1.25–1.46]	0.371
eGFR at 1 year, mL/min/1.73m^2^ (median [IQR])	45.5 [37.0–51.3]	43.6 [36.9–48.3]	0.636
*De novo* DSA during follow-up, n/N (%)*	11/38 (28.9)	1/9 (11.1)	0.412
Graft loss, n (%)	4 (7.4)	0 (0)	0.570
Kaplan–Meier graft survival, log-rank P	—	—	0.191

DSA, donor-specific antibody; IQR, interquartile range; SD, standard deviation; eGFR, estimated glomerular filtration rate. *Evaluable recipients are those with a DSA result (positive or negative) available at any point during follow-up; see Discussion for details of assay methodology and evaluability. **Related donors were parents, siblings, or children; unrelated donors were spouses or other non-blood relatives (e.g., parents-in-law). ***ABO combination (donor→recipient), n (%): A→B, 13 (24.1) vs 4 (26.7); A→O, 10 (18.5) vs. 5 (33.3); B→A, 9 (16.7) vs. 1 (6.7); B→O, 14 (25.9) vs. 3 (20.0); AB→A, 4 (7.4) vs. 2 (13.3); AB→O, 4 (7.4) vs. 0 (0). P value from the Fisher–Freeman–Halton exact test across all six combinations (chi-square test was not used because of small expected cell counts).

## Results

### Patient characteristics

In total, 254 ABOi kidney transplant recipients were identified, of whom 69 met the inclusion criteria and were enrolled in the analysis. Fifty-four recipients (78.3%) were classified into the persistent C4d-positive group and 15 (21.7%) into the persistent C4d-negative/nonpersistent group based on sequential protocol biopsies at 3 months and 1 year post-transplantation. The baseline characteristics are summarized in **Table 1**.

No statistically significant differences were observed between the two groups regarding recipient sex (C4d-positive group: 37/17; C4d-negative group: 11/4; P = 1.000), recipient age (53.1 ± 11.4 vs. 51.7 ± 9.6 years; P = 0.660) and follow-up duration, calculated from the date of transplantation to the last available follow-up (4.8 [1.0–10.8] vs. 4.1 [1.0–9.1] years; P = 0.959). Pre-transplant ABO antibody titers (IgM and IgG) were also comparable between the two groups (IgM: 16.0 [4.0–16.0] vs. 16.0 [4.0–32.0], P = 0.824; IgG: 8.0 [1.0–32.0] vs. 4.0 [1.0–32.0], P = 0.988). Pre-existing DSAs were detected in six patients (11.1%) in the C4d-positive group and one patient (6.7%) in the C4d-negative group (P = 1.000). Donor age, donor relationship to the recipient, and distribution of ABO donor-recipient combinations did not differ significantly between the groups (**Table 1**). Donor sex differed significantly, with a higher proportion of male donors in the C4d-positive group (22/54, 40.7%) than in the C4d-negative group (1/15, 6.7%; P = 0.014).

### Graft outcomes

sCr at 1 year did not differ significantly between the persistent C4d-positive and C4d-negative/nonpersistent groups (1.26 [1.02–1.56] vs. 1.35 [1.25–1.46] mg/dL; P = 0.371), nor did eGFR at 1 year (45.5 [37.0–51.3] vs. 43.6 [36.9–48.3] mL/min/1.73m^2^; P = 0.636). Graft loss occurred in four patients (7.4%) in the C4d-positive group, but none occurred in the C4d-negative group; this difference was not statistically significant (P = 0.570). All four graft losses occurred at 1,849, 1,926, 2,231, and 3,208 d after transplantation. Three of the four (75%) patients ultimately had cABMR, and the fourth had BK virus nephropathy. Notably, one of the three cABMR cases (graft loss at 3,208 days) had biopsy-confirmed cABMR at the 1-year protocol biopsy analyzed in this study (MVI score 3), whereas the remaining two cABMR cases were diagnosed later in the clinical course (1.9 years post-transplant in 1 case; the exact timing could not be retrospectively determined in the other, although the clinical course was consistent with cABMR), at which point no rejection was evident on either the 3-month or 1-year biopsy. A full timeline for each of the four graft-loss cases, including biopsy findings, cABMR diagnosis date, *de novo* DSA detection, graft-loss date, and cause, is provided in **Supplementary Table S2**.

Post-transplant DSA data were available for 47 of the 69 recipients (38 in the C4d-positive group and 9 in the C4d-negative group), while data were unavailable for the remaining 22 recipients. Recipients without available post-transplant DSA data had significantly shorter follow-up than those with available data (median 3.0 vs. 6.0 years; P < 0.001), whereas age, donor age, persistent C4d status, pre-existing DSA, and 1-year eGFR did not differ between the two groups (**Supplementary Table S3**). Among the evaluable recipients, *de novo* DSA detected during follow-up was observed in 11 of 38 (28.9%) patients in the C4d-positive group and in one of nine (11.1%) patients in the C4d-negative group; however, no significant difference was observed between the groups (P = 0.412).

### Banff diagnostic classification of rejection episodes

**Table 2** summarizes the rejection episodes identified in the 3-month and 1-year protocol biopsies, classified according to the Banff criteria. Nine rejection episodes were identified among the 69 analyzed recipients: seven in the persistent C4d-positive group and two in the C4d-negative/nonpersistent group.

**Table 2:** Banff diagnostic classification of rejection episodes identified on the 3-month and 1-year protocol biopsies, by persistent C4d status.

C4d status	Time point	Diagnosis
Persistent C4d-positive (n = 54)	3 months	Borderline change (n = 1)
Persistent C4d-positive (n = 54)	3 months	cABMR (n = 1)
Persistent C4d-positive (n = 54)	1 year	ABMR (n = 1)
Persistent C4d-positive (n = 54)	1 year	cABMR (n = 1)
Persistent C4d-positive (n = 54)	1 year	caTCMR, suspicious (n = 1)
Persistent C4d-positive (n = 54)	1 year	Borderline change (n = 2)
C4d-negative/nonpersistent (n = 15)	3 months	ABMR, suspicious (n = 1)
C4d-negative/nonpersistent (n = 15)	1 year	Chronic active TCMR (n = 1)

Values are numbers of recipients. TCMR, T-cell–mediated rejection; ABMR, antibody-mediated rejection; cABMR, chronic active ABMR; caTCMR, chronic active TCMR.

In the C4d-positive group, at the 3-month biopsy, one case of borderline change and one case of cABMR were observed; at the 1-year biopsy, one case of active ABMR, one case of cABMR, one case of suspected chronic active T-cell-mediated rejection (caTCMR, suspicious), and two cases of borderline change were observed. In the C4d-negative/nonpersistent group, one case of suspected ABMR was observed at the 3-month biopsy, and one case of chronic active TCMR was observed at the 1-year biopsy. Overall, the incidence of rejection remained low in both groups, despite persistent C4d deposition. The case-level histological scores, biopsy-time DSA status, and clinical management for each of the nine rejection episodes are presented in **Supplementary Table S4** to allow a direct assessment of the basis for each diagnosis.

### Association of C4d positivity with histological scores

The relationship between persistent C4d positivity and Banff histological scores was assessed at 3 months and 1 year post-transplantation (**Figures 3**–**5**). For sensitivity analysis, the same comparisons were repeated using a stricter, Banff-concordant definition of persistent positivity (C4d score ≥ 2 at both time points; n = 47 positive, n = 22 negative), since a C4d score of 1 (minimal) is not conventionally regarded as positive under the Banff criteria (**Supplementary Figure 3-1S** to **3-3S**).

**Figure 3:**
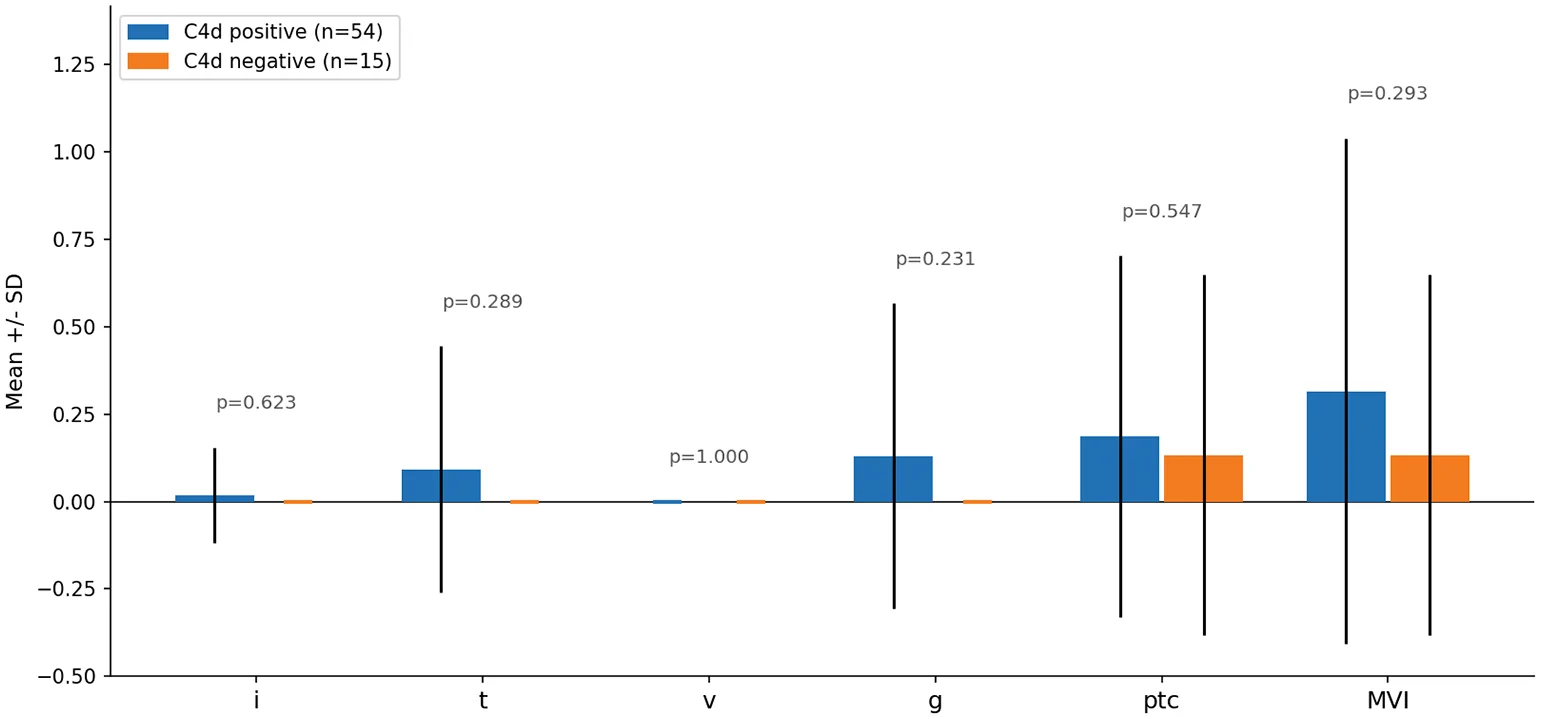
Acute lesion scores at the 3-month protocol biopsy (mean ± SD) in the persistent C4d-positive group (n = 54) and the C4d-negative/nonpersistent group (n = 15). i, interstitial inflammation; t, tubulitis; v, intimal arteritis; g, glomerulitis; ptc, peritubular capillaritis; MVI, microvascular inflammation (g + ptc). No significant differences were observed between the groups (all P > 0.05). A sensitivity analysis using the stricter Banff-concordant definition of persistent positivity (C4d score ≥ 2 at both time points; n = 47 positive, n = 22 negative) is shown in **Supplementary Figure 3-1S** and likewise showed no significant differences.

**Figure 4:**
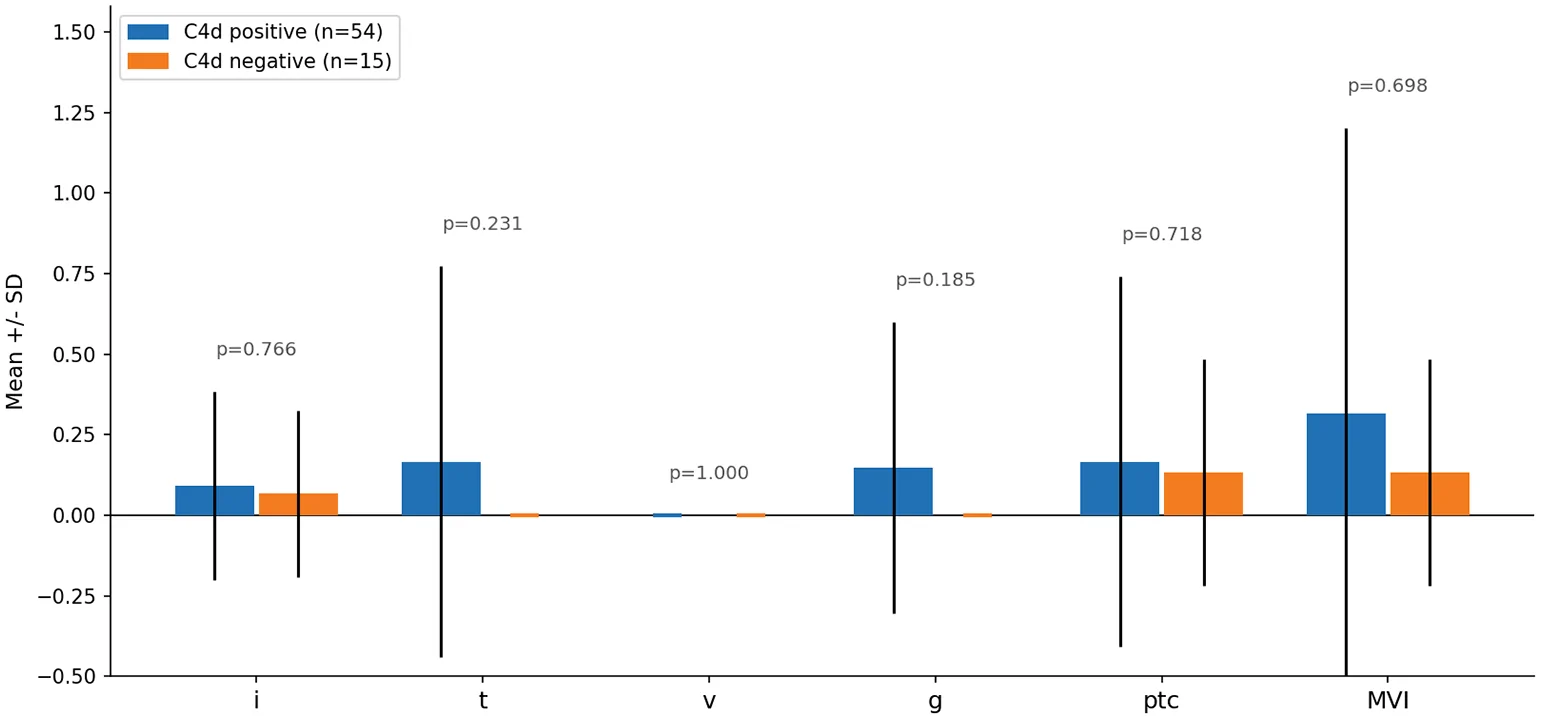
Acute lesion scores at the 1-year protocol biopsy (mean ± SD) in the persistent C4d-positive group (n = 54) and the C4d-negative/nonpersistent group (n = 15). Abbreviations as in **Figure 3**. No significant differences were observed between the groups (all P > 0.05). Corresponding sensitivity analysis in **Supplementary Figure 3-2S**.

**Figure 5:**
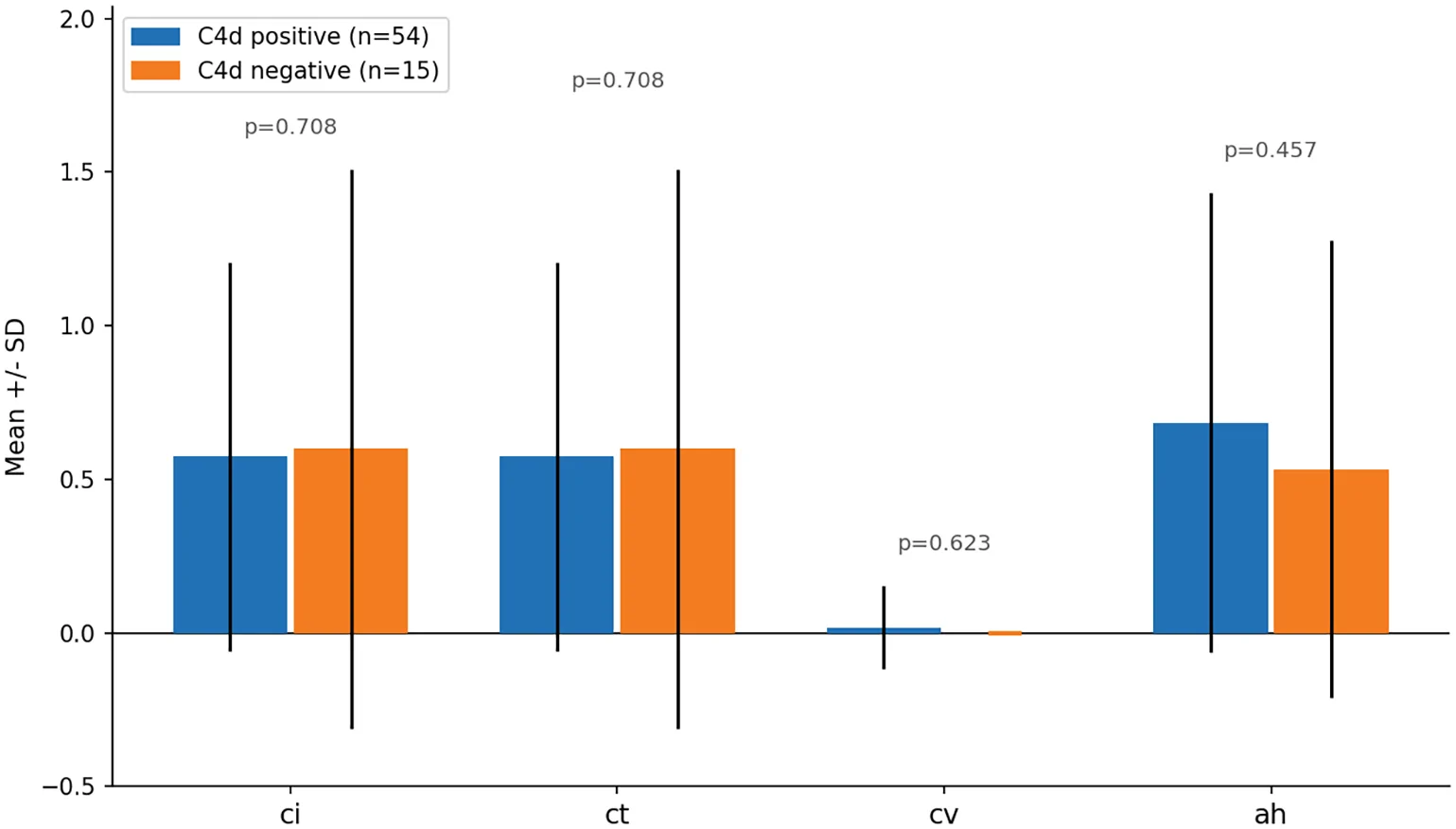
Chronic injury scores at the 1-year protocol biopsy (mean ± SD) in the persistent C4d-positive group (n = 54) and the C4d-negative/nonpersistent group (n = 15). ci, interstitial fibrosis; ct, tubular atrophy; cv, vascular intimal thickening; ah, arteriolar hyalinosis. No significant differences were observed between the groups (all P > 0.05). Corresponding sensitivity analysis in **Supplementary Figure 3-3S**.

At the 3-month protocol biopsy (**Figure 3**), cellular infiltration scores, including g, ptc, i, and t, were compared between C4d-positive and C4d-negative recipients.

Cellular infiltration scores were similarly evaluated at the 1-year protocol biopsy (**Figure 4**).

Chronic injury scores at 1 year (**Figure 5**), including ci, ct, cv, and ah, were also examined.

### Graft survival

Kaplan–Meier analysis showed no significant difference in graft survival between C4d-positive and C4d-negative recipients (**Figure 6A**; log-rank test, P = 0.191). Because group assignment requires a 1-year protocol biopsy, all analyzed recipients are by definition event-free survivors for up to 1 year. To address the resulting guarantee-time bias, the analysis was repeated as a landmark analysis with time origin at the 1-year protocol biopsy. This conclusion remained unchanged in the landmark analysis (**Figure 6B**; log-rank test, P = 0.191).

**Figure 6:**
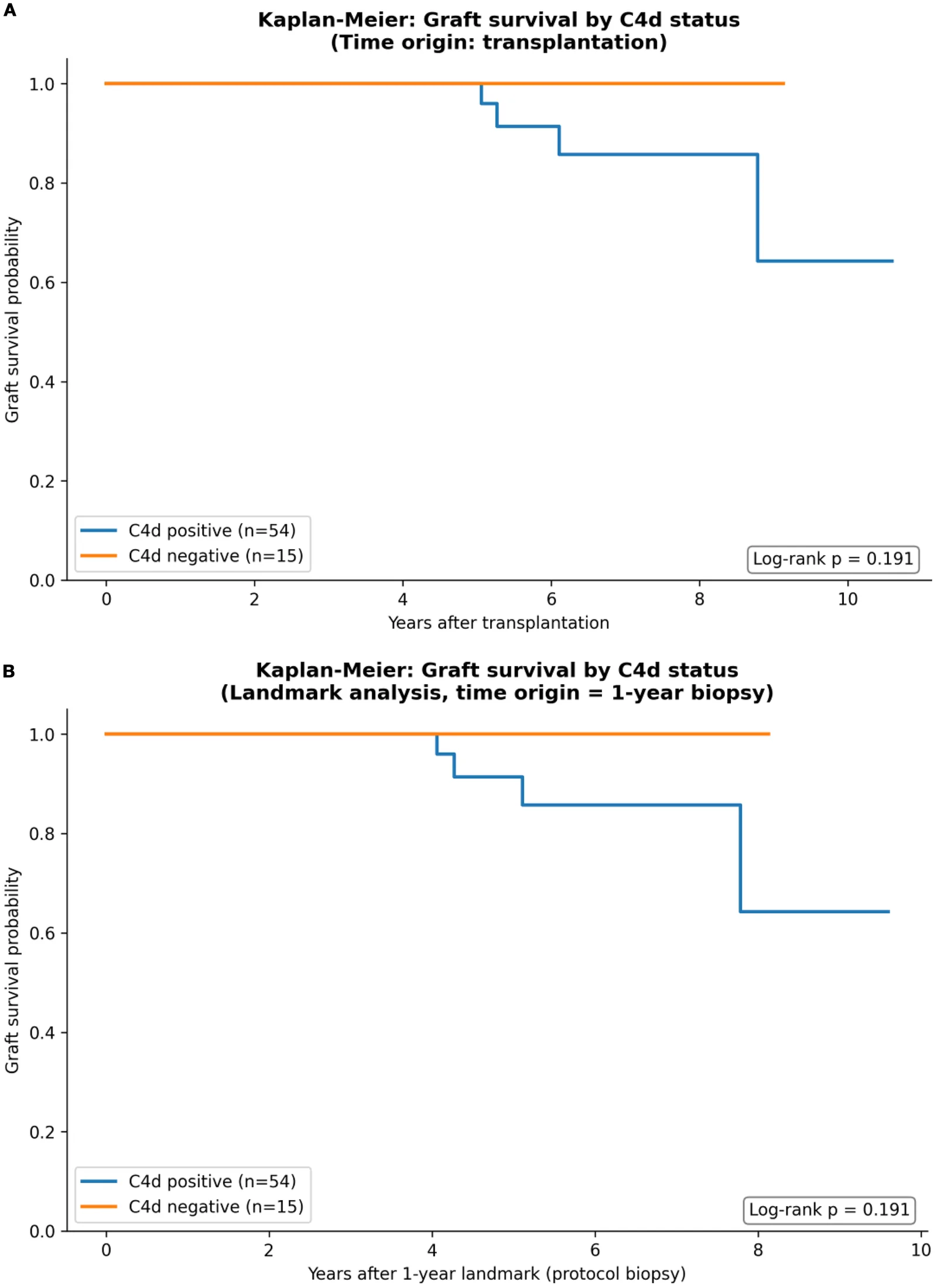
Kaplan–Meier estimates of graft survival according to C4d status. **(A)** Time origin at transplantation. **(B)** Landmark analysis with time origin at the 1-year protocol biopsy, addressing guarantee-time bias arising from the requirement of a 1-year biopsy for group assignment. Graft survival did not differ significantly between the persistent C4d-positive group (n = 54) and the C4d-negative/nonpersistent group (n = 15) in either analysis (log-rank P = 0.191 for both). All four graft-loss events occurred in the C4d-positive group; no events occurred in the C4d-negative group.

## Discussion

In this study of ABOi kidney transplant recipients, persistent C4d deposition detected on sequential post-transplant protocol biopsies at both 3 months and 1 year was not associated with recipient pre-transplant background (anti-A/B antibody titers, ABO combination, or pre-existing anti-HLA antibodies) or, on those same post-transplant biopsies, with cellular infiltration or MVI or with the development of chronic Banff lesions. No statistically significant differences in graft function or graft survival were observed, although all graft losses in the analyzed cohort occurred in the persistently C4d-positive group. To our knowledge, most previous studies on ABOi transplantation have evaluated C4d at a single time point. The principal contribution of the present work is to define C4d positivity by its persistence across two sequential protocol biopsies and to examine whether this reproducible, time-stable pattern carries the prognostic weight that isolated C4d positivity has been assumed to lack.

The near-ubiquity of C4d deposition after ABOi transplantation, together with its apparent clinical silence, is consistent with the concept of accommodation (6, 8): membrane-bound complement regulatory proteins such as CD46, CD55, and CD59 restrain complement-mediated injury on the graft endothelium despite ongoing antibody binding, providing a plausible mechanistic basis for the histological pattern observed in the present cohort (7, 12, 13).

Against this background, the low incidence of rejection in both groups is notable. Despite persistent C4d deposition, biopsy-proven rejection in the C4d-positive group was infrequent, and no association was observed between persistent C4d and cellular infiltration at either time point. Because the difficulty of diagnosing ABMR in ABOi transplantation stems largely from the high background frequency of C4d positivity, MVI (g plus ptc) was specifically evaluated, following the observation of Ishihara et al. (14) that MVI is more informative than C4d in this setting; the Banff 2022 consensus has since reaffirmed the central diagnostic role of MVI and of biopsy-based transcript diagnostics (15). MVI scores did not differ between C4d-positive and C4d-negative recipients at either time point, and chronic Banff lesions (ci, ct, cv, and ah) at 1 year were similar. sCr levels at 1 year, graft loss, and Kaplan–Meier graft survival (**Figures 6A, B**; log-rank P = 0.191 for both) also showed no significant differences. Although graft survival did not differ significantly, it is noteworthy that all four cases of graft loss in the analyzed cohort occurred in the persistent C4d-positive group. Whether this numerical imbalance reflects a genuine, subtle effect of persistent C4d deposition on long-term outcomes, or simply the absence of events in a small C4d-negative group of only 15 patients, cannot be resolved with the present sample size and warrants confirmation in larger cohorts. Taken together, at the group level, persistent C4d deposition was not associated with a higher burden of concurrent histological injury during the first post-transplant year, although, as discussed further below, individual recipients with persistent C4d did go on to develop overt humoral injury later in their clinical course.

These findings extend, rather than contradict, earlier work from our center. Setoguchi et al. (16) reported that diffuse C4d positivity, observed in approximately 60% of ABOi protocol biopsies, had little diagnostic or therapeutic relevance. Ishihara et al. (14) emphasized MVI over C4d for graft deterioration. Similarly, Cho et al. (17) linked peritubular capillary C4d to outcomes only when it co-occurred with MVI, and recent molecular data further supported the distinction between C4d deposition due to accommodation and true alloimmune injury in ABOi grafts. Comparative protocol-biopsy studies by Masutani et al. (18) and Dørje et al. (19) found broadly similar allograft pathology between ABO-compatible and ABOi grafts. Our data reinforce these findings and add to the observation that even reproducible, persistent C4d, arguably the pattern most likely to reflect a genuine ongoing process, is not accompanied by the inflammatory or chronic changes that Mengel et al. (20) proposed as morphological correlates of ongoing alloimmune injury.

A recent single-center study by Lopez et al. reported similar findings (21). In that study, which compared ABOi and ABO-compatible allografts, C4d deposition in ABOi grafts was interpreted as a marker of adaptation, rather than active injury. Although anti-A/B isoagglutinins are donor-directed antibodies, they were not used in this study to satisfy the Banff serological criteria for ABMR. Furthermore, the authors proposed that molecular diagnostics might help distinguish true immune-mediated injuries from adaptation. Our findings of persistent C4d deposition without MVI, chronic injury, or graft dysfunction are consistent with this view.

One finding warrants cautious emphasis rather than dismissal. As described in the Methods section, post-transplant DSA testing was not performed at a uniform time point in this retrospective cohort (available measurements were obtained between approximately 4 months and 3 years after transplantation), and *de novo* DSA was therefore evaluated as detection at any point during follow-up rather than at a fixed 1-year time point. Using this definition, *de novo* DSA was detected in 11 of 38 evaluable recipients (28.9%) in the persistent C4d-positive group, compared with one of nine (11.1%) in the C4d-negative group. Post-transplant DSA data were unavailable for the remaining 22 recipients; retrospective verification could not reliably distinguish between absence of testing and unavailability of historical test results, and we could not confirm that this missingness was unrelated to recipient characteristics; a comparison of recipients with versus without evaluable DSA data is provided in **Supplementary Table S3**, which showed significantly shorter follow-up among recipients without available post-transplant DSA data, but no significant difference in age, donor age, C4d status, pre-existing DSA, or 1-year eGFR. The frequency of *de novo* DSA (28.9% vs. 11.1%) did not reach statistical significance (P = 0.412). This numerical difference is exploratory and cannot be interpreted as evidence of increased *de novo* DSA frequency in the persistent C4d-positive group because DSA surveillance timing and follow-up duration were non-uniform across recipients, as detailed above. Whether persistent C4d deposition is unrelated to subsequent HLA sensitization or reflects low-grade humoral activity that could contribute to later sensitization cannot be determined from this retrospective, non-uniformly surveyed cohort. The absence of molecular transcript profiling further limited our ability to detect subclinical humoral activity, which may distinguish between these possibilities. Prospective studies with systematic, uniformly timed DSA surveillance and molecular assessments are required to determine whether persistent C4d expression is associated with long-term immunological risk.

A related observation is that all four graft losses in this cohort occurred in the persistently C4d-positive group. Three of the four (75%) cases were ultimately attributable to cABMR, and the fourth to BK virus nephropathy. Notably, these three cABMR cases were not homogeneous with respect to the timing of diagnosis relative to the protocol biopsies analyzed in this study. One recipient (graft loss at 3,208 days) had biopsy-confirmed cABMR at the 1-year protocol biopsy used to define C4d persistence in this study, with an MVI score of 3. Early persistent C4d was accompanied by concurrent histological injury, as captured by our assessment. In the remaining two cABMR cases, and in the case attributable to BK virus nephropathy, no MVI or overt rejection was present on the 3-month or 1-year biopsies; cABMR was diagnosed later in the clinical course (1.9 years post-transplant in 1 case; the exact timing could not be retrospectively determined in the other). This heterogeneity indicates that persistent C4d at 3 months and 1 year does not reliably distinguish recipients who will remain stable from those who will later develop overt humoral injury and that the absence of concurrent histological injury at these early time points does not exclude later risk. This observation is consistent with and extends the cautious interpretation of the *de novo* DSA findings above and suggests that long-term immunological surveillance, rather than either reassurance or immediate treatment escalation based on early biopsies alone, may be warranted in recipients with persistent C4d in the first year.

One incidental finding warrants brief comment. Donor sex differed significantly between groups, with a substantially higher proportion of male donors among C4d-positive recipients than among C4d-negative recipients (40.7% vs. 6.7%, P = 0.014). We are not aware of a mechanistic basis linking donor sex to C4d persistence and given the number of baseline comparisons performed in this study, this finding may represent a chance association. It was reported for completeness rather than as a substantive result, and it should be interpreted with caution pending replication in larger cohorts.

This study had several limitations. First, this was a retrospective, single-center study. Second, and most consequentially, the cohort was small, particularly the C4d-negative/nonpersistent comparator group (n = 15); thus, the study was substantially underpowered for low-frequency clinical endpoints, such as rejection episodes and graft loss. The absence of statistically significant differences in this setting is likely to reflect a type II error rather than true equivalence and should be interpreted as a failure to detect an effect rather than as evidence that no effect exists; several comparisons (e.g., graft loss, 4/54 vs. 0/15) have correspondingly wide confidence intervals. Therefore, we cannot determine whether persistent C4d deposition is clinically relevant.

Third, the analyzed cohort represented a highly selected subgroup of the overall ABOi population. Only 69 of the 254 recipients were analyzed, and the cohort was deliberately restricted by excluding recipients with treated rejection prior to protocol biopsy (n = 19), TMA (n = 12), IVIG at induction (n = 37), retransplantation, or recurrent glomerular disease to isolate the effect of C4d from these complement- and injury-modifying factors. A further 96 recipients were excluded because paired protocol biopsies with sufficient pathological material were unavailable at both 3 months and 1 year, and the majority (n = 91) reflected biopsies performed outside the intended 3-month/1-year protocol window, including scheduling delays and interruptions related to the COVID-19 pandemic, and 5 reflected patient refusal of the biopsy procedure. These exclusions effectively removed the higher-risk population, and the corollary was a selection bias that may have precisely removed recipients in whom C4d deposition coincided with clinical injury. Therefore, our findings should be understood as applicable to a highly selected cohort of clinically stable recipients with paired protocol biopsies and adequate pathological material rather than to the broader ABOi transplant population, and our null result should be read in that light.

Fourth, anti-A/B antibody titers were available only before transplantation; titers at or near the time of the 3-month and 1-year biopsies were not systematically measured. Without contemporaneous antibody data, we could not determine whether persistent C4d deposition reflected ongoing exposure to circulating donor blood group-specific antibodies with successful accommodation or merely residual complement footprints in the absence of such antibodies. Therefore, our interpretation of accommodation was provisional.

Fifth, C4d was assessed semi-quantitatively by immunofluorescence, and the biological significance of low-grade (score 1) deposition remains uncertain; to address this, we repeated all comparisons using a stricter, Banff-concordant definition of persistent positivity (C4d score ≥ 2 at both time points), which yielded a materially larger C4d-negative comparator group (n = 22) and did not change the overall pattern of findings (**Supplementary Figure 3-1S** to **3-3S**). Antibody titers at the time of each rejection episode, where available, are reported at the case level in **Supplementary Table S4** rather than stratified into predefined titer categories because the range of recorded values was too narrow to support meaningful stratification into higher-titer strata. Sixth, C4d assessment was performed by a single renal pathologist who was not blinded to the clinical information. Formal inter-observer reproducibility was not assessed because C4d scoring was performed by the same pathologist throughout the study period. These factors may have introduced observer-related bias into the assessment of C4d deposition. Seventh, molecular diagnostics such as biopsy-based transcript profiling (e.g., the Molecular Microscope Diagnostic System) were not available for this cohort. As transcriptomic assessment is increasingly used to distinguish true subclinical ABMR from accommodation and is emphasized in the Banff 2022 report (15), the absence of molecular data limits our ability to exclude low-grade ongoing humoral activity that is not captured by conventional histology. Eighth, the available follow-up data limit inferences regarding long-term graft survival, which should, therefore, be interpreted with caution. Finally, multiple histological comparisons were performed without adjusting for multiplicity; although none were significant, this further supported a conservative interpretation.

## Conclusion

In conclusion, in this small, highly selected cohort of clinically stable ABOi kidney transplant recipients with paired protocol biopsies, persistent C4d deposition on sequential 3-month and 1-year protocol biopsies was not associated with recipient pre-transplant background, cellular infiltration, MVI, or the development of chronic Banff lesions. No statistically significant difference was observed in graft function or graft survival, although all four graft losses occurred in the C4d-positive group; three were ultimately attributable to chronic active ABMR, diagnosed at the 1-year protocol biopsy in one case and years later in the remaining two cases. This heterogeneity raises the possibility that long-term immunological surveillance may be informative in recipients with persistent C4d deposition, even in those without concurrent histological injury at 3 months or 1 year. However, this cannot be established from the present observational data. Given the degree of cohort selection and the limited statistical power, these findings should not be extrapolated to a broader ABOi transplant population, nor should they be interpreted as establishing that persistent C4d deposition lacks clinical relevance. These observations are hypothesis-generating and require confirmation in larger, prospective cohorts with longer follow-up periods and systematic *de novo* DSA surveillance.

## Data Availability

The datasets generated and/or analyzed during the current study are not publicly available because they contain potentially identifiable clinical information. De-identified data may be made available by the corresponding author upon reasonable request, subject to approval by the relevant institutional review board and institutional data-sharing policies.

## Supplementary material

Supplementary Figure 3-1S Acute lesion scores at the 3-month protocol biopsy (mean ± SD), sensitivity analysis using the stricter, Banff-concordant definition of persistent C4d positivity (C4d score ≥ 2 at both time points; n = 47 positive, n = 22 negative). i, interstitial inflammation; t, tubulitis; v, intimal arteritis; g, glomerulitis; ptc, peritubular capillaritis; MVI, microvascular inflammation (g + ptc). No significant differences were observed between the groups (all P > 0.05).

Supplementary Figure 3-2S Acute lesion scores at the 1-year protocol biopsy (mean ± SD), sensitivity analysis as in Supplementary Figure 3-1S (n = 47 positive, n = 22 negative). Abbreviations as in Supplementary Figure 3-1S. No significant differences were observed between the groups (all P > 0.05).

Supplementary Figure 3-3S Chronic injury scores at the 1-year protocol biopsy (mean ± SD), sensitivity analysis as in Supplementary Figure 3-1S (n = 47 positive, n = 22 negative). ci, interstitial fibrosis; ct, tubular atrophy; cv, vascular intimal thickening; ah, arteriolar hyalinosis. No significant differences were observed between the groups (all P > 0.05).
